# Workforce Mobilization From the National Institutes of Health for the Ministry of Health Malaysia: A COVID-19 Pandemic Response

**DOI:** 10.3389/fpubh.2021.574135

**Published:** 2021-02-11

**Authors:** Abdul Rassip Muhammad Nur Amir, Awatef Binti Amer Nordin, Yin Cheng Lim, Nor Izzah Binti Ahmad Shauki, Nor Hayati Binti Ibrahim

**Affiliations:** ^1^Institute for Health Management, Ministry of Health Malaysia, Shah Alam, Malaysia; ^2^Institute for Health System Research, Ministry of Health Malaysia, Shah Alam, Malaysia; ^3^Institue for Medical Research, Ministry of Health Malaysia, Shah Alam, Malaysia

**Keywords:** COVID-19 pandemic, data management, Ministry of Health Malaysia, National Institutes of Health Malaysia, workforce mobilization

## Abstract

The COVID-19 pandemic that emerged in 2019 has inflicted numerous clinical and public health challenges worldwide. It was declared a public health emergency by the World Health Organization and activated response teams at almost all Malaysian healthcare facilities. Upon activation of the National Crisis Preparedness and Response Center in January 2020, the National Institutes of Health Malaysia established a COVID-19 operation room at the facility level to address the rise in COVID-19 infection cases each day. The National Institutes of Health COVID-19 operation room committee formed a workforce mobilization team for an effective and efficient mobilization system to fulfill requests received for human resource aid within the Ministry of Health Malaysia facilities. Selected personnel would be screened for health and availability before mobilization letters and logistics arrangements if necessary. The workforce from the National Institutes of Health, consisting of various job positions, were mobilized every week, with each deployment cycle lasting 2 weeks. A total of 128 personnel from the six institutes under the National Institutes of Health were mobilized: tasks included fever screening, active case detection, health management at quarantine centers, and management of dead bodies. A well-organized data management system with a centralized online system integration could allow more rapid deployment and answer some of the key questions in managing a similar pandemic in the future. With improving infected COVID-19 cases throughout the country, the National Institutes of Health COVID-19 operation room was effectively closed on June 15, 2020, following approval from the Deputy Director-General of Health.

## Introduction

COVID-19 is an infectious disease caused by a newly discovered coronavirus that reportedly emerged in Wuhan city, China, in December 2019. This novel coronavirus's causative agent was in January 2020 found to be of the same subgenus as the Severe Acute Respiratory Syndrome Coronavirus (SARS-CoV). As of September 17, 2020, there were a total of 29,656,504 COVID-19 cases worldwide, with 936,905 deaths reported (1). The World Health Organization (WHO) declared COVID-19 a pandemic on March 11, 2020, making it a public health emergency of international concern (2). Cases have been reported virtually in all continents and have been steadily increasing in many countries. The three countries most impacted by COVID-19 include the United States of America (USA), which reported cases to surpass 6 million, followed by India and Brazil, with more than 9 million combined cases. On September 17, 2020, Malaysia had more than 10,000 confirmed cases of COVID-19, with 128 deaths and among the highest number of coronavirus infections in Southeast Asia (3).

This ongoing outbreak poses many clinical and public health management challenges because of limited understanding of viral pathogenesis; infection risk factors; disease clinical presentation and outcomes; prognostic factors for severe illness; the infectivity period; modes and range of virus inter-human transmission; effective preventive measures as well as public health response; and containment interventions (4). Extra efforts need to be in place to prevent and control the spread of an infectious disease or pandemic. The healthcare system changes during a pandemic, whereby shortages of medical personnel, staff illnesses, or limitations in the availability of equipment or intensive-care unit capacity becoming the major triggering factors (5).

The first wave of COVID-19 outbreak in Malaysia began with the emergence of the first confirmed case detected on January 24, 2020. Up until mid-February, Malaysia recorded 22 cases of COVID-19. The Ministry of Health (MOH) Malaysia activated the National Crisis Preparedness and Response Center (CPRC) in January 2020 under the Surveillance Section of the Disease Control Division to ensure effective management of disasters, outbreaks, crises, and emergencies (DOCE) related to health. After the second wave of COVID-19 in Malaysia, which occurred in late February 2020, the MOH Malaysia recognized a need for workforce reinforcements, essentially targeted screening within the community and International Points of Entry. Hence, the CPRC aims to provide short-term relief and strategize on mobilizing ancillary medical and health personnel along with volunteers from the MOH Malaysia, government and non-governmental bodies, private sectors, and individuals as part of the public health response to areas with the highest burden of COVID-19.

## The Sudden Surge

Malaysia was able to report 11 days of zero confirmed cases consecutively, from February 16 to 26, 2020, until the second wave began February 27, 2020. On March 11, 2020, Malaysia experienced a sharp increase in new cases after the International Health Regulations (IHR) Focal Point for Brunei told the Malaysian IHR Focal Point that the country had discerned a positive case traveling to Malaysia to attend a religious mass meeting. The meeting was held from February 27 to March 1, 2020, involving approximately 16,000 participants, of which about 14,500 participants were Malaysians. More than 100 cases have been registered daily since March 15, 2020, with most patients having a history of attending the mass meeting or some manner of contact with the attendees (6). Subsequently, on March 17, 2020, Malaysia recorded the first two COVID-19-related deaths, with one case being associated with the religious gathering.

In mid-March, the rampant outbreak suggested that Malaysia was in the late containment phase and required swift and more aggressive measures to contain the disease. Consequently, the Malaysian government instituted a Movement Control Order (MCO) from March 18 to March 31, 2020, intending to break the community's COVID-19 chain. The MCO was further extended in phases until May 3, 2020, as the number of positive cases remained relatively high. In many specific locations that reported a sudden rise in cases, the Malaysian government introduced Enhanced MCO (EMCO). With Conditional MCO (CMCO) (May 4 to June 9, 2020) and Recovery MCO (RMCO) (June 10 to August 31, 2020) implemented sequentially, the four-phase MCO has nearly flattened the curve and decreased the active cases to an amount that does not overwhelm the healthcare system.

## Context – Setting and Population

### Coordination of Country Response

Malaysia initiated a response to COVID-19 as soon as the news broke of a new pneumonia-causing virus in Wuhan, China, on January 2, 2020. After corresponding with the WHO Country Office as well as the WHO Regional Office, Malaysia's IHR focal point shifted to evaluating the risk of occurrence in Malaysia. Subsequently, on January 5, 2020, Malaysia's IHR focal point began collecting information through the WHO Events Information Site (EIS), a safe web-based communications portal between the WHO and the National IHR focal point. *Via* the EIS, the WHO Secretariat exchanged information and communication regarding acute public health risks with potentially global ramifications. Based on the information and risk assessment provided by the WHO-EIS, Malaysia's IHR focal point conveyed the information to the Director-General of Health, all Deputy Directors of Health, relevant program directors in the MOH, as well as other relevant ministries, such as the Department of Veterinary Affairs. Then, an urgent meeting of the Disaster Management Technical Committee took place to coordinate Malaysia's preparedness and response.

Malaysia's IHR focal point provides leverage in its response by frequently communicating with the National IHR focal points from other countries. In due course, on January 23, 2020, Malaysia's IHR Focal Point received an email from Singapore's IHR focal point regarding the need for contact tracing of the first COVID-19 case in Singapore. These led to Malaysia's first COVID-19 event, which was identified as one of the contacts from Singapore's case 2 days later, January 25, 2020. Effective communication between IHR focal points enabled early identification and quarantine to reduce Malaysia's disease spread. On the same day, the Malaysian IHR focal point notified the WHO of the first case of COVID-19 in Malaysia using IHR (2005) Annex 3. Malaysia is currently still reporting its cases weekly *via* the WHO website: https://COVID-19-dataentry-who.hub.arcgis.com.

Several national policies have been developed as guidelines in matters relating to crisis and security. One such guideline is Directive 20 of the National Security Council, which includes defined frameworks for the command, control, and coordination of health emergency preparedness and response. These frameworks are facilitated by the National Disaster Management Agency (NADMA) under the National Disaster Management Committee. This directive outlines the routes of response, roles, and obligations of each relevant agency, such that in the event of a health crisis, the MOH leads the technical direction. Several other laws and policy documents were also applicable to the response to COVID-19, including the Infectious Disease Prevention and Control Act (Act 342), the International Health Regulations, 2005 (IHR, 2005), and the MOH Disaster Management Plan (MOH, 2015).

**Figure 1:**
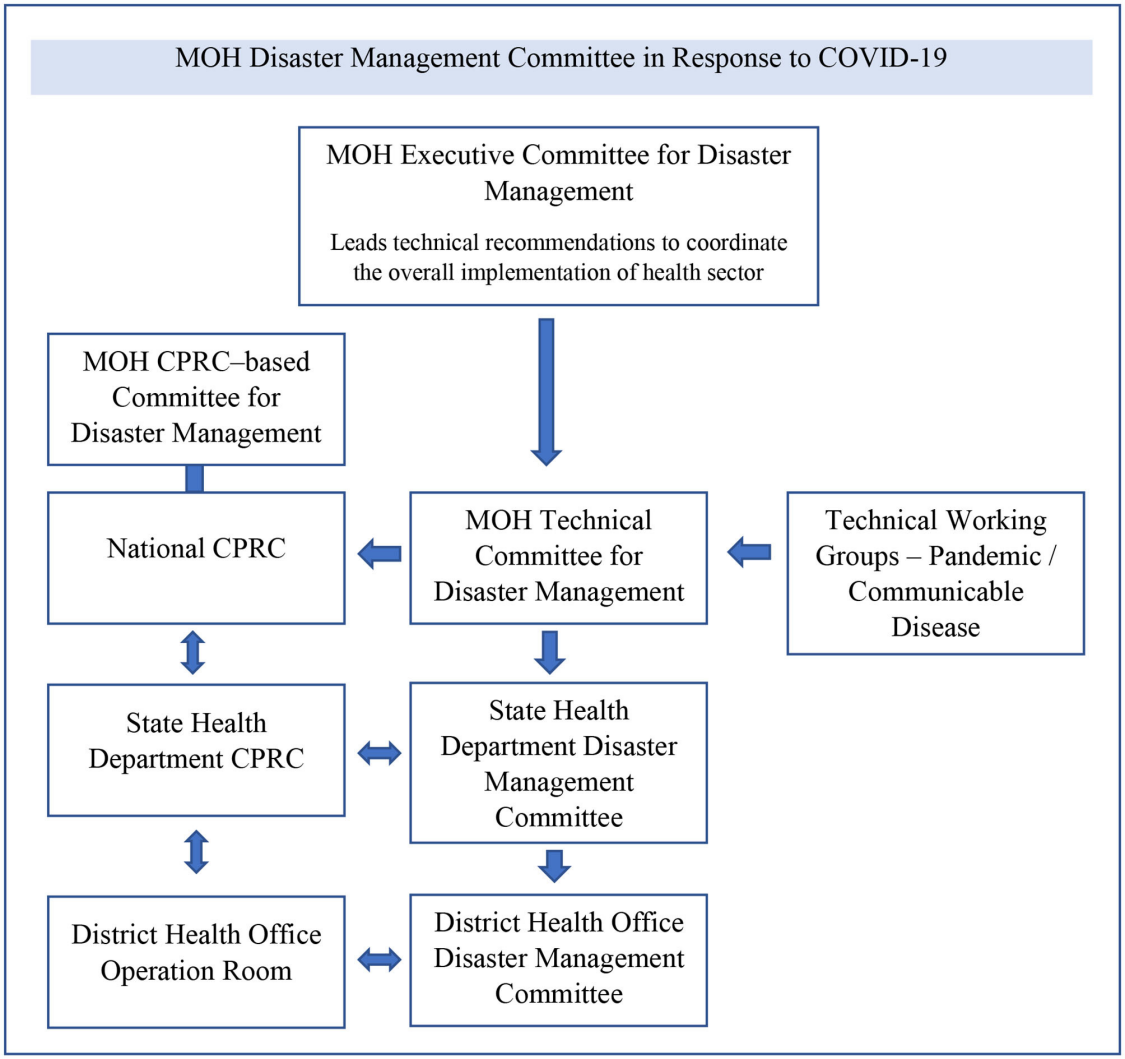
MOH disaster management committee in response to COVID-19. (Source: MOH, MOH Disaster Response Plan, 2015).

The National Security Council (NSC), which works through committees at national, state, and district levels, manages the execution of overall policies involving multiple agencies. The national center for public health emergency operations supervised the planning, coordination, and implementation of response activities. It is known in Malaysia as the National Crisis Preparedness and Response Center (CPRC), MOH. The MOH provides technical advice through its disaster response committees, including the national CPRC (Figure 1). The national CPRC cascades the flow of command to propagate hospital services and primary care responses by central-level coordinators and is ultimately enforced at state and district levels.

## Workforce Mobilization Team

The second wave of COVID-19 in Malaysia has precipitated the call for human resources reinforcements to support the District Health Offices' implementation of public health operations and containment measures. Contact tracing of the participants of the mass religious gathering was done actively by the district health teams, and the hardest-hit areas were the states of Selangor and Kuala Lumpur. Furthermore, the national CPRC, through its MOH COVID-19 Mobilization Support Unit, has prepared, organized, and enforced the deployment of human resources and medical countermeasures in the region. Simultaneously, with border control measures, the auxiliary workforce was also needed to screen travelers at international entry points, such as Kuala Lumpur International Airport. Hence, it was a requisite for MOH to strategize on the mobilization of healthcare workers and volunteers in affected areas where an additional workforce was required. The number of workforces, working hours, and each team's composition to be deployed was focused on the State Health Departments' needs and demands. The State Health Departments regularly tracked the progress of activities within each state.

A need assessment was carried out to determine the requirements of human resources during the pandemic. With the information obtained from field implementation activities and consideration of other factors, like the increasing number of cases, the national CPRC evaluated the gaps between existing conditions and the resources needed. These factors are summarized below.

### Evaluation of Surge Capability

Exponential estimates of the number of cases and close contacts, increased R-naught, increased laboratory processing time, decreased data reporting timeliness, inadequate data quality, and overworked ground workers were indicators of an overwhelmed public health response system. The state health departments have identified limitations in human resources to the national CPRC, which has taken steps to overcome shortages.

Several factors also contributed to the rise in human resource requirements. For instance, the rapid increase of COVID-19 cases in the second wave created a massive patient burden, adding enormous strain to the current healthcare workers. In circumstances where healthcare workers had themselves contracted COVID-19, and their duties were suspended, the workforce shortages were exacerbated. All of them had to undergo COVID-19 testing and were quarantined for a fortnight. Furthermore, the Low-Risk COVID-19 Treatment and Quarantine Centers' opening scattered around Malaysia also required additional medical human resources.

### Assessment of The Deployment Scope of Activities

Another relevant point considered in the healthcare personnel deployment was to assess the extent of the tasks to be carried out in maintaining the existing essential health services in MOH facilities and deal with increased workloads due to the handling of COVID-19 cases. The scope of the activities of healthcare personnel deployed are listed in the Table 1.

**Table 1:** Scope of activities for the healthcare personnel during deployment.

**No**.	**Scope of activities**
1	Identifying of active cases
2	Assisting the monitoring of PUI home surveillance
3	Health monitoring of the organization employees
4	Issuing Quarantine Order Forms
5	Distributing PPE
6	Providing a service for psychosocial assistance
7	Swab sampling of symptomatic and asymptomatic patients
8	Managing data
9	Providing orientation and training sessions for volunteers
10	Assisting frontliners in the care of patients
11	Carrying out interviews with contract-for-service staff
12	Hotline call duties
13	Chest X-ray / Laboratory testing
14	Producing PPE and preparation that includes aprons, face mask, headgear, boot cover, etc.
15	COVID-19-related research
16	Transporting COVID-19 PUI and supporting patients to the respective health facilities
17	Determining health workers for mobilization
18	Supervising sites and providing input on any inquiries made by volunteers
19	Assisting at the State and District Health Operations Centers
20	Contact tracing of the PUI from various sources and notifications
21	Determining the need for and organizing mobilization team accommodation
22	Preserving current essential healthcare services
23	Supplying first aid and medical care to volunteers (when necessary)
24	Individual and mass screening
25	Aiding public inquiries related to COVID-19
26	Managing the deceased in cases of COVID-19

Priority is given for the MOH deployment, whereby personnel recruitment was made through communication between national and state CPRCs. Personnel involved in the mobilization of COVID-19 activities dependent on the tasks' feasibility and nature. The COVID-19 pandemic brought a response from all government agencies, for instance, the Ministry of Defense, the Ministry of Housing and Local Government, and the Ministry of Human Resources, enrolling masses of their staff to assist public health teams. The MOH has also obtained assistance from medical professionals and volunteers from the private and civil society (non-governmental organizations or NGOs) who have met the same recruitment requirements (7).

## National Institutes of Health COVID-19 Operation Room

The National Institutes of Health (NIH) COVID-19 operation room was established on March 17, 2020, following the activation of national CPRC and mandated by the Deputy Director-General of Health to help deal with the sudden surge of COVID-19 cases in Malaysia. The NIH is a unique healthcare organization under the MOH Malaysia consisting of six different institutes in a shared compound with a workforce of around 1,300 and various job positions from different medical/health backgrounds. The NIH is a tessellation of the Institute for Medical Research, Institute of Public Health, Institute for Health Management, Institute of Health System Research, Institute of Health Behavior Research, and Institute for Clinical Research. The NIH's core businesses include health-related research, conducting or organizing training and courses, and offering consultancy for the Ministry of Health personnel (8).

The NIH COVID-19 operation room serves as the center for communication and coordination of all activity relating to control and prevention of COVID-19 in NIH while harmonizing activity as well as the need from the national CPRC and State Health Departments. Besides anticipating requests from other healthcare facilities, the operation room is also responsible for the health surveillance of NIH personnel involved in the control and prevention of COVID-19 activity. Health promotion activities regarding COVID-19 for NIH personnel, including concession companies attached with NIH (e.g., cleaning services, security, catering), are coordinated by the NIH COVID-19 operation room.

As one of the NIH core businesses is research, all research activities relating to COVID-19, including diagnostic laboratory, are monitored by the NIH COVID-19 operation room. Other functions of the NIH COVID-19 operation room encompass the focal center for strategic cooperation between ministries and agencies in managing the spread of the COVID-19 pandemic and identifying and coordinating the need for accommodations for the workforce mobilization team.

In ensuring a smooth and effective mobilization system, a workforce mobilization team lead by the NIH COVID-19 operation room committee was formed with assistance from each institute's liaison officers under the NIH, which had been discerned beforehand. Liaison officers posited to identify personnel and prepare a list according to personnel readiness for mobilization and location preferences. Since activation from March until June 2020, the NIH COVID-19 operation room have received human resource aid requests from the national CPRC, Selangor State Health Department, and Federal Territory of Kuala Lumpur/Putrajaya Health Department as early as 2 weeks before mobilization *via* email or telephone.

Subsequently, selected personnel need to be under 60 years of age, be registered as a licensed medical practitioner in Malaysia with the Malaysian Medical Council to conduct tasks involving contact with patients, and agree to sign a liability document. They would then be screened for health, availability, and clearance from the supervisor before a letter of mobilization can be issued and logistic arrangements made if necessary.

In the early phase of the COVID-19 pandemic, human resources in Malaysia's healthcare sectors focused on detecting and quarantine COVID-19 cases (8). Hence, other divisions that were not involved in early detection activities, including the NIH, offered human resource aid to other healthcare counterparts on the ground as frontliners. However, mobilized personnel from the NIH could be called back to work immediately upon their supervisors' request, together with substantial justification.

**Figure 2:**
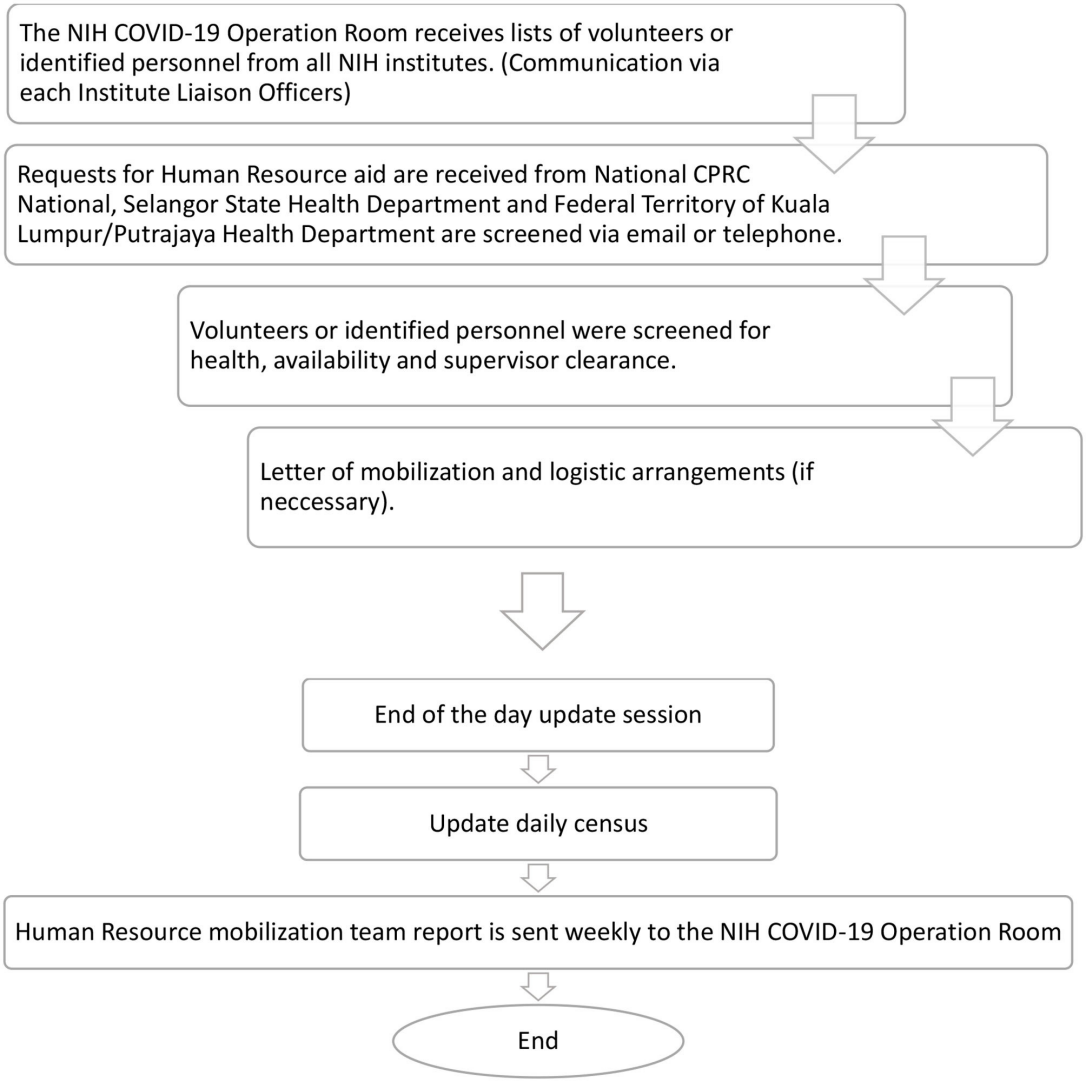
The workflow for the COVID-19 operation room NIH mobilization team.

As the need for an extra workforce increased, more NIH personnel volunteered to assist. Teams were mobilized every week, but each cycle of deployment lasted 2 weeks. The number of personnel and composition of each deployment team was based on the requests of the national CPRC and State Health Departments. A pre-deployment briefing was given for each batch of groups, while further training was carried out at the assigned locations. Each briefing covered administrative matters, job scope, infection prevention, control, as well as accommodation etiquette. The workflow of facilitating mobilization is visualized in Figure 2.

## Details To Understand Key Programmatic Elements

The first deployment began with eleven personnel to the national CPRC and Sungai Buloh Hospital (the main COVID-19 treating hospital) on March 17, 2020. Later, on March 29, 2020, the number quadrupled to 47 mobilized personnel to the national CPRC, Selangor State Health Department, three district health offices in Selangor, and Sungai Buloh Hospital. Throughout the establishment of the NIH COVID-19 operation room, it is estimated about 128 personnel were cumulatively mobilized from every institute under the roof of NIH to ease the burden on another healthcare workforce due to the COVID-19 pandemic. All six institutes actively provided human resource aid to the MOH facilities around the Federal Territory of Kuala Lumpur & Putrajaya and the largest state by population, Selangor. Considering those who volunteered and those who were assigned for attachment for career advancement, Table 2 shows the location and the total number of personnel mobilized from the NIH.

**Table 2:** Location and the total number of mobilized personnel from the National Institutes of Health Malaysia.

**No**.	**Location**	**Total mobilized**
1	National Crisis Preparedness and Response Center (CPRC), Ministry of Health	44
2	Selangor State Health Department	14
3	Federal Territory of Kuala Lumpur & Putrajaya Health Department	14
4	Petaling District Health Office	20
5	Gombak District Health Office	6
6	Hulu Langat District Health Office	8
7	Sepang District Health Office	6
8	International Entry Points, Kuala Lumpur International Airport (KLIA)	4
9	Kuala Lumpur General Hospital	5
10	Sungai Buloh Hospital	7
Total		**128**

The human resource mobilization team received involvement from every job position level, which depicted a high level of teamwork and a sense of responsibility within the MOH during this challenging period. Public health physicians, medical officers, dental officers, environmental health officers, and nurses have valuable skills and clinical expertise that can be appropriately used to assist frontliners. Public health physicians and environmental health officers are also the most competent to conduct public health measures under the Prevention and Control of Infectious Disease Act 1988 (Act 342), such as issuing a quarantine order. Other job positions were determined later on, according to where this group of different backgrounds and skilled personnel could be best stationed. The breakdown of personnel mobilization by job position is shown in Table 3.

**Table 3:** Number of mobilized personnel by job positions.

**No**.	**Job position**	**Total mobilized**
1	Public Health Physicians	6
2	Medical Officer	60
3	Research Officer	21
4	Dental Officer	1
5	Health Education Officer	4
6	Science Officer	5
7	Environmental Health Officer	1
8	Nurse	4
9	Pharmacy	6
10	Statistician	4
11	Assistant Research Officer	1
12	Assistant Statistician	2
13	Assistant Environmental Health Officer	1
14	Administrative Assistant	9
15	Assistant Librarian	3
Total		**128**

As the human resource aid that we provided began to relieve the pressure and load from the assigned locations, other job positions were sent for non-clinical support. The non-clinical support mainly evolves around data management as data received daily could be overwhelming for small operation centers like the district offices. After the first month of personnel mobilization, most clinical personnel have returned to their respective institutes to continue the existing tasks. The movement of the entire NIH personnel was put into a master list using Microsoft Excel. As a result, a live database of mobilized workforces was created and accessible to all NIH personnel for monitoring as well as record keeping. This database was also being used as health surveillance and staff attendance, which are salient in emergencies.

## Discussion

The national CPRC was established in January 2020 by the Ministry of Health Malaysia and triggered activation of CPRC and COVID-19 operation room at other healthcare facilities around the country, abiding by the Event-based Surveillance Protocol in addressing the public health emergency of international concern (9–11). The human resource mobilization division for the national CPRC was founded on March 11, 2020. Their first deployment team comprised 60 personnel from seven State Health Departments to the Selangor state health department and the Federal Territory of Kuala Lumpur health department. This human resource mobilization team's underlying motivation and aim were to efficiently mobilize the healthcare workforce as a COVID-19 response in a strategic, safe, astute, and resource-conscious way.

Our response team's efforts in identifying evolving needs and promptly engaging relevant workforces within the MOH had countless advantages. Many non-clinical workforces were keen to combat the deadly pandemic but felt powerless. However, research has shown that united action toward a common goal can parry these emotions (12). Together with enthusiasm toward such a desire, orderly measures can foster a sense of empowerment, motivation, and connection. An effective disease control strategy demands an efficient public health workforce, sophisticated integrated public health and clinical health information technology, trained human resources, and substantial community involvement (4, 12).

A similar response observed in South Africa, where the Africa Centers for Disease Control and Prevention has initiated an African Task Force for Coronavirus Preparedness and Response (ATFCOR) focusing on six workstreams: laboratory diagnosis and subtyping; surveillance (including screening at points of entry and cross-border activities); infection prevention and control in healthcare facilities; clinical treatment and management of people with severe COVID-19; risk communication; as well as supply chain management and stockpiles (13).

The effectiveness of an organization's human resources management and its team development is a significant factor that affects that organization (14). The human resource mobilization team in the NIH COVID-19 operation room act as a coordinator for personnel deployment from NIH and require inter-organization commitment as well as necessitate full responsibility from the liaison officers for an effective system. All six NIH institutes had their respective organization and sub-organization that potentially delayed the process of verifying personnel for mobilization. The situation was exacerbated when requests were received for human resource aid at the latest possible time resulting in engendering personnel to prepare for mobilization inadequately. Regardless, the mobilized team was briefed to equip them mentally and physically, albeit tasks were given by assigned medical facilities independently. There is precedent for such an enormous mobilization to address a pandemic. In reducing the spread of Ebola, African countries mobilized thousands of caseworkers while China reportedly mobilized 18,000 public health workforces to curb the spreading of COVID-19 in Wuhan alone (15).

To help in response to COVID-19, other countries, such as the USA and Croatia, mobilized their medical students to do anything from providing childcare to healthcare workers to enrolling in short-term roles in the healthcare system (13–15). Malaysia utilizes a different approach whereby, instead of recruiting medical students, we encourage volunteers from retirees with a medical background. We also received volunteers from other ministries such as the Ministry of Housing and Local Government and Malaysian Armed Forces; non-government organizations (NGO) like Medical Relief Society Malaysia, Malaysia Relief Agency, and Islamic Medical Association of Malaysia Response & Relief Team; as well as government agencies, for example, the Department of Occupational Safety and Health Malaysia. These collaborations with NGOs and other agencies, resulting in a seamless distribution of the workforce, food supplies, and personal protective equipment (PPE) all around Malaysia. Moreover, the collaborations provide an opportunity for medical personnel outside of the MOH to contribute and serve the country.

As a result of lessons learned during the SARS and influenza pandemic, the development and standardization of plans, including human resource mobilization, operational support, space, and equipment, are vital in the first few hours of an outbreak. Deployment and mobilization should be assessed early and frequently during the response to enhance the preparedness of pre-identified workforces (16). The AsiaFluCap Project (2008–2011), which was based in Germany, analyzed the feasibility and capability of health system resources mobilization across selected Asian countries in the event of an influenza pandemic. Using a mathematical transmission model, they could estimate gaps in healthcare resources vital for responding to the influenza pandemic by simulating a mild to moderate scenario of an influenza pandemic (17). Lesson learnt from that exercise, suggesting that a pandemic could have a high public health impact with the maldistribution of resources.

Our mobilized team received recognition nationally for their contribution to the country. Apart from offering a helping hand to the overwhelming workload, we have also developed new methods to manage the COVID-19 pandemic, which were adapted by the MOH Malaysia. For instance, an electronic form was created for a comprehensive data collection on contact tracing. The template has been used in several states in Malaysia along with a density map that inspires modeling with a projection of COVID-19 virus spread based on daily cases and reproduction number (18, 19).

Nevertheless, the human resource mobilization team faced a few challenges in the course of its operation. Receiving an overwhelming amount of volunteers since the inception of the NIH COVID-19 operation room, the process of screening these volunteers was time-consuming and calling up identified volunteers labor-intensive. The lack of a medical volunteer registry to recruit personnel slowed the process of deployment in the beginning. There were also several challenges with regards to the deployed healthcare personnel's skills and competencies. Throughout the early phases of the pandemic and with COVID-19 being a new disease, healthcare personnel may not have been entirely aware of or familiar with the clinical recommendations for the treatment of COVID-19.

Moreover, the personnel themselves were at constant risk of exposure to the virus. This risk is exacerbated by the threat of psychological consequences such as anxiety and depression due to stigma. It was essential to track the well-being of all deployed personnel through a system of continuous guidance and close supervision as well as monitoring by the occupational health and safety teams within healthcare facilities. Well-established mental health and psychosocial support platforms were also crucial in ensuring the continued morale and desire of deployed personnel to fulfill their duties.

Another realization that arose during the pandemic is related to all deployed personnel's readiness to work in a crisis setting. The COVID-19 challenged the healthcare system to such an extent that all staff, from technical to support and clinical to non-clinical, were entailed in some way in the country response, thus coining terms such as “frontliners” and “backliners.” Many deployed personnel had steep learning curves to quickly familiarize themselves with the given tasks that were very different from the routine day-to-day work and in a full of urgency environment. Tasks have been carried out with involvement from all organizations, both governmental and non-governmental, depicting an all-out approach from a large team. Therefore, all personnel need to have some sort of readiness training in a public health emergency setting, and these preparations should be not only physical but also mental. The ability to predict potential challenges to healthcare personnel during a pandemic crisis would add value in ensuring more effective and productive management for future crises.

One of the workforce mobilization teams' unforeseen challenges was the growing numbers of infected healthcare personnel, which has resulted in a shortage of personnel in some hospitals. Human resources for health became scarce, and considerations were required to ensure healthcare quality and delivery uninterrupted. As a result, a range of measures has been outlined to ensure the continuation of health services. First of all, priority was given to emergency departments and hospital wards regarding the distribution of resources. Mobilization of healthcare personnel from other facilities, either interstate or intrastate, and personnel rotation between departments were among the measures taken. In certain situations, the Occupational Safety and Health (OSH) Units of the health facilities ordered the temporary closure of those departments that have had a few healthcare personnel with positive COVID-19. The whole department was disinfected and sanitized before the resumption of operations (7).

As a proactive response, the MOH introduced and implemented various ground-breaking concepts, such as teleconsultations, drive-thru pharmacies, and notifications of waiting numbers *via* messages or WhatsApp®. These improvisations helped to minimize the need for healthcare personnel to be physically present and indirectly limit the interaction and exposure between the healthcare personnel and patients. In the event of severe staff shortages, the OSH officer will assess the healthcare personnel to decide the appropriateness for an early return to work.

The NIH COVID-19 operation room recognized there are some possible limitations from the workforce mobilization activity. Firstly, mobilizations were based on request and availability at a particular moment. Hence, the assessment of support needed and given may not necessarily reflect the operation's actual requirements. Indeed, the whole experience has been a lesson in teamwork and coordination. The different strengths of the respective personnel in the various teams have harmoniously complemented each other by creating a diverse group that has responded potently against this pandemic situation.

This article also focuses on the workforce mobilization from the NIH to the other public healthcare facilities within the MOH Malaysia in the early phases of the COVID-19 pandemic management in Malaysia. The requests that we received only come from the central region. The Crisis Preparedness and Response Center, MOH Malaysia, based in the Federal Territory of Putrajaya, coordinates workforce mobilization involving interstates and other healthcare facilities. However, the lack of information, especially on average working hours of the volunteers and the work shift system implemented at individual locations, impede estimating the optimal number of personnel to be mobilized. Instead, the workforce mobilization is varying and is subjectively tailored to the request from the locations mentioned earlier. These variables are valuable to improve the efficiency of mobilization in the future.

## Conclusion

Mobilization of human resources within the MOH was a critical technique to maximize human resource capacity during a pandemic. Developing a database of volunteers ready for future deployment in a disaster or crisis may become crucial in human resource mobilization management. The next step should be establishing an online volunteer management system that would integrate the current database and allow for more rapid deployment. Such a centralized system would ease the recruitment process in terms of accurately identifying the available expertise and locating suitable experts to be deployed to selected regions. A carefully established data management and relevant information availability might be the key to answering some of the challenges faced during pandemic management in the future, including COVID-19.

The NIH COVID-19 operation room was effectively closed on June 15, 2020, succeeding the Deputy Director-General of Health's approval after observing improved infected COVID-19 cases throughout the country. Nonetheless, the war against the COVID-19 pandemic demands an efficient workforce and effective response team that can rise to the challenge for a safer and healthier world.

## Data Availability Statement

The original contributions presented in the study are included in the article/supplementary material, further inquiries can be directed to the corresponding author/s.

## Author Contributions

AM was the Head of the Workforce Mobilization committee for NIH COVID-19 Operation Room. The NIH COVID-19 Operation Room was led by NIB as the commander and assisted by YC as the deputy commander. The operation room would not be successful without AB as the coordinator and supervised the NIH personnel movement database. AM, YL, AB, and NIB contributed tremendously to the production of this manuscript. NHB reviewed and gave technical advisory toward the manuscript as well as contributed essential revisions. All authors read and approved the manuscript.

## Conflict of Interest

The authors declare that the research was conducted in the absence of any commercial or financial relationships that could be construed as a potential conflict of interest.

## Abbreviations

ATFCOR: African Task Force for Coronavirus Preparedness and Response
COVID-19: Coronavirus Disease 2019
CPRC: Crisis Preparedness and Response Center
DOCE: Disaster, Outbreak, Crises, and Emergencies
EIS: Events Information Site
IHR: International Health Regulations
MOH: Ministry of Health
NADMA: National Disaster Management Agency
NGO: Non-Government Organization
NIH: National Institutes of Health
NSC: National Security Council
OSH: Occupational Safety and Health
PPE: Personal Protective Equipment
PUI: Patient Under Investigation
SARS-CoV: Severe Acute Respiratory Syndrome Coronavirus 2
USA: United States of America
WHO: World Health Organization.
